# Overworked School-Girls

**Published:** 1875-06

**Authors:** 


Overworked School-Girls. — The serious evil in connection with many public schools, is not so much the forgetfulness of sex in education as the forgetfulness of sex in home training and social life. Girls would be much less depressed by their school studies if their afternoons and evenings were not constantly devoted to other and exacting duties. Their music-lessons twice a week, dancing-school lessons as often, together with social visiting, occasion of themselves a fearful strain upon the nervous system, and utterly unfit girls for their studies. The whole tone of some schools is seriously affected by the constant absence, during a portion of the school hours, of the young pupils, both boys and girls. They are taking lessons in music and dancing, and are excused from school nearly one-half of the hours of instruction, and, through the necessary home practice upon the piano, become so much wearied as to be quite unfitted for study. There can be no more effectual way to break down the system, and to prevent a healthy and happy mental growth and cultivation on the part of the child, than such a course as this. Music and dancing may be safely delayed until greater physical and mental development have been secured; and, in thousands of instances, even mental development should be delayed until a vigorous out-door life shall have given to the constitution something like permanent vitality.

There is another view of this matter also. These public schools are sustained at great expense, and parents that avail themselves, without cost, of all their facilities, should feel a moral obligation to meet the moderate requisitions they make upon the pupil's time. The State making all this gratuitous provision has a right to claim attendance on the part of children, and to be defended from any unnecessary interruption of its beneficial designs. Sensible and conscientious parents, if they permit themselves to think candidly upon this subject, will soon convince themselves of the wrong they are doing, both to the schools and their children, in thus constantly demanding their absence to cultivate prematurely, and at the expense of more important studies, the ornamental branches of education. Healthy children have all the demand upon their nervous energy that they should be required to put forth, in the regular calls of their daily studies. When the health is somewhat delicate, the injury resulting from these additional drafts is even more serious.

